# Recombinant Human BMP6 Applied Within Autologous Blood Coagulum Accelerates Bone Healing: Randomized Controlled Trial in High Tibial Osteotomy Patients

**DOI:** 10.1002/jbmr.4107

**Published:** 2020-07-02

**Authors:** Catharina Chiari, Lovorka Grgurevic, Tatjana Bordukalo‐Niksic, Hermann Oppermann, Alexander Valentinitsch, Elena Nemecek, Kevin Staats, Markus Schreiner, Carmen Trost, Alexander Kolb, Franz Kainberger, Sanja Pehar, Milan Milosevic, Snjezana Martinovic, Mihaela Peric, T Kuber Sampath, Slobodan Vukicevic, Reinhard Windhager

**Affiliations:** ^1^ Department of Orthopedics and Trauma Surgery Medical University of Vienna Vienna Austria; ^2^ Laboratory for Mineralized Tissues, Centre for Translational and Clinical Research University of Zagreb School of Medicine Zagreb Croatia; ^3^ Genera Research Rakov Potok Croatia; ^4^ Department of Environmental and Occupational Health and Sports, School of Public Health, “Andrija Stampar,” University of Zagreb School of Medicine Zagreb Croatia; ^5^ SmartMedico Zagreb Croatia; ^6^ Department for Intracellular Communication, Centre for Translational and Clinical Research University of Zagreb School of Medicine Zagreb Croatia; ^7^ perForm Biologics Inc. Holliston MA USA

**Keywords:** BMP, CLINICAL TRIALS, IMPLANTS, INJURY/FRACTURE HEALING

## Abstract

Bone morphogenetic proteins (BMPs) are potent osteogenic proteins that induce new bone formation *in vivo*. However, their effect on bone healing in the trabecular bone surfaces remains challenging. We evaluated the safety and efficacy of recombinant human BMP6 (rhBMP6) applied within an autologous blood coagulum (ABC) in a surgically created wedge defect of the proximal tibia in patients undergoing high tibial osteotomy (HTO) for varus deformity and medial osteoarthritis of the knee. We enrolled 20 HTO patients in a randomized, placebo‐controlled, double‐blinded phase I/II clinical trial. RhBMP6/ABC (1.0 mg/10 mL ABC prepared from peripheral blood) or placebo (10 mL ABC containing excipients) was administered into the tibial wedge defects. Patients were followed for 0 to 24 months by clinical examination (safety) and computed tomography (CT) and serial radiographic analyses (efficacy). The results show that there were no detectable anti‐rhBMP6 antibodies in the blood of any of the 20 patients at 14 weeks after implantation. During the 24 months of follow‐up, there were no serious adverse reactions recorded. The CT scans from defects of patients treated with rhBMP6/ABC showed an accelerated bone healing compared with placebo at 9 weeks (47.8 ± 24.1 versus 22.2 ± 12.3 mg/cm^3^; *p* = 0.008) and at 14 weeks (89.7 ± 29.1 versus 53.6 ± 21.9 mg/cm^3^; *p* = 0.006) follow‐up. Radiographic analyses at weeks 6 and 24 and months 12 and 24 suggested the advanced bone formation and remodeling in rhBMP6/ABC‐treated patients. In conclusion, we show that rhBMP6/ABC at a dose of 100 μg/mL accelerated bone healing in patients undergoing HTO without serious adverse events and with a good tolerability compared with placebo alone. Overall, for the first time, a BMP‐based osteogenic implant was examined against a placebo for bone healing efficacy in the trabecular bone surface, using an objective bone mineral density measurement system. © 2020 The Authors. *Journal of Bone and Mineral Research* published by American Society for Bone and Mineral Research.

## Introduction

Bone heals completely by itself upon fracture. However, among smokers and steroid users, metabolic insults like diabetes and osteopenia, as well as infection after commuted open fractures, bone healing is compromised and remains challenging. Recombinant human bone morphogenetic proteins (rhBMPs) have been used to promote bone healing in patients with acute open fractures and delayed and non‐union diaphyseal shaft fractures. Recombinant human bone morphogenetic protein 2 (rhBMP2) and 7 (rhBMP7) in conjunction with bovine collagenous matrix as carrier have been used in long bone fractures, but their effect remains controversial in metaphyseal regions of cancellous bone (hip, tibia, and wrist) fractures.^(^ ^1^, ^2^, ^3^, ^4^, ^5^, ^6^, ^7^ ^)^ In trabecular bone surfaces, application of rhBMPs has been shown to induce osteolysis of native bone, swelling, redness, and delayed bone healing,^(^ ^1^, ^8^ ^)^ likely attributed to the high dose of rhBMPs and use of animal‐derived collagen as a carrier. Because cancellous bone injuries are most prevalent, an rhBMP‐based biological therapy with a physiologically acceptable carrier would be beneficial to patients.

We recently described a novel autologous bone graft substitute (ABGS) that is composed of rhBMP6 applied within autologous blood coagulum (ABC).^(^ ^9^ ^)^ ABGS induced new bone formation at 20 to 100 μg rhBMP6/mL coagulum in rat subcutaneous implants and restored the diaphyseal segmental defects in preclinical models.^(^ ^9^, ^10^ ^)^ ABC serves as a non‐immunogenic physiological carrier. Furthermore, BMP6 has higher specific osteogenic activity than BMP2 and BMP7 presumably because it does not bind irreversibly to Noggin, a BMP antagonist, abundant in bone, compared with BMP2 and BMP7.^(^ ^6^, ^7^, ^11^ ^)^

In the present study, we assessed intraosseal administration of ABGS, ie, rhBMP (rhBMP6) loaded within autologous blood coagulum, in adult patients who underwent a high tibial osteotomy (HTO). HTO is a surgical procedure performed to correct varus malalignment of the lower limb in order to delay the progression of medial osteoarthritis of the knee.^(^ ^12^, ^13^, ^14^, ^15^, ^16^ ^)^ HTO involves creating a wedge defect mainly in the proximal metaphyseal region of the tibia for which iliac crest autograft is used to accelerate bone healing and regeneration,^(^ ^17^ ^)^ but it is not preferred.^(^ ^18^ ^)^ This study was a randomized, double‐blind, placebo‐controlled phase I/II trial. The primary objective of the study was to assess safety, tolerability, anti‐BMP6 antibody response, if any, and systemic pharmacokinetics (PK) of the application 100 μg rhBMP6/mL ABC locally into the wedge gap after osteotomy. The secondary objective was to assess the acceleration of bone healing in the wedge gap. Here, we evaluated for the first time the effects of ABGS (treatment) and ABC with excipients (placebo) in HTO patients,^(^ ^18^, ^19^, ^20^ ^)^ initially followed by computed tomography (CT) scans and then radiographically during the course of healing.

## Materials and Methods

### Intervention

The investigational therapy in the trial was rhBMP6 within ABGS, which was compared with placebo (PBO) as reference therapy. Allocated treatment (ABGS) was delivered locally into the HTO osteotomy defect as a single dose. In addition to intervention, all patients received the standard of care. The source of rhBMP6 and PBO (excipients only) was Genera Research Ltd (Rakov Potok, Croatia), which provided the investigational medicinal product kit that has been manufactured, tested, and released into the trial according to GMP principles.

The ABGS and PBO implants were prepared using patient's blood collected from the cubital vein at least 90 minutes before time of dosing. In a closed sterile cabinet located in the surgical ward as described,^(^ ^7^ ^)^ ABGS was prepared with 1.0 mg of rhBMP6 at a concentration of 100 μg/mL using 10 mL blood. PBO was prepared using 10 mL blood, where only excipients were added and was provided as a blinded control to the surgeon. The final therapy was prepared in a syringe and formed a paste‐like, uniformly homogeneous, and cohesive implant for surgical administration.

The open‐wedge HTO is a surgical technique to correct varus malalignment of the lower limb, caused by a deformity of the tibia.^(^ ^13^ ^)^ After osteotomy, a wedge is opened leaving a bone gap and the defect site is stabilized using a locking plate. In all patients an arthroscopy of the knee was performed to document the cartilage status and, if necessary, to perform minor concomitant procedures such as meniscal debridement. The operation procedure is described in detail in Supplemental Materials and Methods.

### Clinical study design

The study was designed as a randomized, double‐blinded, placebo‐controlled phase I/II clinical trial to evaluate the safety and efficacy of ABGS (rhBMP6/ABC) and PBO administered in the tibia wedge‐defect gap of patients who elected to undergo HTO procedure. The study was conducted at one site in Austria (Medical University of Vienna, Department of Orthopedics and Trauma Surgery) under trial registration number EudraCT 2015–001691‐21. The total number of patients enrolled was 20 with a final assignment ABGS/PBO 1:1, 6 included in phase I and 14 in phase II. Phase I was intended to obtain “first‐in‐human” safety data on a total of 6 patients, 5 of whom were randomized to ABGS and 1 to PBO. Details of the clinical study design can be found in the Supplemental Materials and Methods, and the full trial protocol can be assessed at https://www.clinicaltrialsregister.eu/ctr‐search/trial/2015‐001691‐21/AT.

### Sample size determination

Based on historical data, the dynamics of HTO defect healing in the placebo group was expected to average around 20% at week 9 post‐surgery, around 40% at week 14 post‐surgery, and approximately 90% after 6 months.^(^ ^21^ ^)^ The sample of 20 patients (ABGS/PBO randomized in 1:1 ratio) was selected to provide 80% power to detect an average difference between ABGS and PBO of around 30% in defect healing. The bone‐healing acceleration effect was estimated using X‐ray analyses according to the established radiological scoring system,^(^ ^16^, ^22^ ^)^ and the bone mineral density (BMD), which reflected the bone formation in the defect area, was measured by CT scans using ITK‐SNAP program.^(^ ^16^, ^23^ ^)^ This clinical model allows estimation of new bone formation within a bone defect protected by a locking plate to eliminate possible influence of mechanical stimuli. As healing was expected in all cases, no end points of mechanical failure have been determined. Further details about the sample size determination are indicated in Supplemental Materials and Methods.

### Inclusion and exclusion criteria

Patients for the HTO study were included if they had a symptomatic varus deformity and had self‐reported good or excellent health. A signed informed consent was obtained before any study assessment was performed. Both males and females at age ≥18 years were included. Females of childbearing potential were negative in urine pregnancy test before the randomization. None of the patients included in the study had received platelet aggregation inhibitors at least 5 days before the surgery. Patients were excluded from the study if they had any of the following: previous osteotomies of the tibia; inflammatory and neoplastic diseases of the knee joint; previous treatment with rhBMPs; clinically significant hepatic disease or other abnormalities in screening laboratory tests; an uncontrolled medical condition, including bone metabolic (in particular osteoporosis and medical history of treatment with anti‐osteoporotic drugs), renal, endocrine, hepatic, respiratory, cardiovascular, hematologic, immunologic or cerebrovascular disease, and malignancy; history of symptomatic nephro‐ or urolithiasis within 2 years; diabetes mellitus; treatment with an investigational drug within 6 months preceding the first dose of study medication; positive urine pregnancy test; breastfeeding a child or planning to become pregnant within 6 months; use of non‐steroid anti‐inflammatory drugs (NSAIDs; paracetamol accepted) and systemic corticosteroids except for dexamethasone for the first 2 days postoperatively; evidence of human immunodeficiency virus (HIV) antibody; hepatitis B infection within the past year or history of non‐adequately treated hepatitis C infection; drug or alcohol abuse; donation of blood in excess of 500 mL within 56 days before and 1 month after surgery; and current participation in any other clinical trial. The patients provided written informed consent before any study procedure and the trial was approved by the Austrian competent authorities.

### Safety outcome measures

Safety was assessed continuously throughout the trial based on clinical signs, serial vital signs assessments, laboratory assessments, and spontaneously reported adverse events. Local safety/tolerability was specifically assessed by clinical inspection (eg, signs of inflammation), pain and functional assessment, as well as radiological assessment with a particular focus on possible soft tissue ossification. Additionally, serum samples were taken for anti‐rhBMP6 antibodies analysis from all patients before drug administration and at week 14 and stored at −80°C until analysis. The samples were analyzed using a validated indirect ELISA system for anti‐rhBMP6 antibodies, according to the recommendations described in the literature.^(^ ^24^ ^)^ As additional safety monitoring, in the first 24 hours, blood samples were collected for PK assessment. Further details regarding safety monitoring and outcome measures can be found in Supplemental Materials and Methods.

### Efficacy outcome measures

Bone‐healing acceleration was assessed using CT and radiographic analysis. In terms of the safety assessment, X‐ray images taken at week 6 were used for detection of the occurrence of potential soft tissue ossifications. Postoperative CT assessment was conducted at weeks 9 and 14. The CT data were used to measure the acceleration of early bone healing after treatment. X‐ray assessment was used at 24 weeks and at months 12, 18, and 24 to monitor long‐term bone healing. Efficacy outcome was defined as percentage of defect filled with newly formed bone, based on CT assessments performed at weeks 9 and 14 after surgery and X‐ray analyses of day 1, weeks 6 and 24, and months 12, 18, and 24. The BMD, as well as bone formation in the defect area, was measured on CT scans by ITK‐SNAP program performed at weeks 9 and 14 post‐surgery (designed and validated at the University of Pennsylvania and University of Utah, 2014^(^ ^23^ ^)^). Detailed description of BMD estimation can be found in Supplemental Materials and Methods.

Bone healing in the wedge‐gap defect was estimated based on X‐ray analyses according to the published radiological scoring system.^(^ ^22^ ^)^ For evaluation, the defect was divided into four zones numbered from lateral to medial, as shown in Fig. 5. In each zone, the area of unfused osteotomy was measured and presented as percentage of filled bone, as previously described.^(^ ^25^ ^)^

### Study oversight

The HTO study was performed as part of the OSTEOGROW^(^ ^6^ ^)^ consortium effort and investigators who were not employed by the study sponsor (Genera Research) oversaw the execution of the protocol and planned the analyses before unblinding the treatment assignments. The sponsor held the data and performed the analyses and the academic authors received all analyses that they requested. The sponsor designed the protocol and was responsible for the management and quality control.

The important changes to the initial trial protocol included: extension of the follow‐up period from initial 6 to 24 months to enable the evaluation of the potential late safety outcomes and introduction of 18‐month interim analysis of clinical and radiological findings.

### Randomization and blinding

The randomization was implemented off‐site (at Contract Research Organization [CRO]) during the product labeling, ensuring the random allocation of treatment as per trial protocol (ABGS/PBO: phase I 5:1, phase II 5:9, respectively) using a simple randomization method. The treatment and the placebo could not be distinguished because both vials and their contents as well as the final implants were of the same visual appearance. The participants were enrolled into the trial by study investigators and allocated to treatment group by means of sequentially numbered, opaque, and sealed envelopes (SNOSE) stored in a secure container. The patients, investigators, site personnel, and the pharmacist were blinded to the treatment assignment. The blinded pharmacist randomized participants after eligibility confirmation, prepared all treatments based on randomization, and was responsible for securing the randomization envelopes in a secure location. Data analysis was performed by blinded evaluators.

### Statistics

All statistical analyses are described in detail in the Supplemental Materials and Methods.

### Study approval

The clinical study protocol was approved by the Medical University of Vienna, Department of Orthopedics and Trauma Surgery Ethics Committee (EK‐No 1349/2015) and by the Austrian regulatory agency BASG/AGES (BASG reference 8967915). All participants provided written informed consent before their inclusion in the study and for the prolonged study follow‐up. The study was conceived, designed, initiated, and performed by the academic investigators. The authors confirm the accuracy and completeness of the data and analysis and the fidelity of the study to the protocol. All the authors agreed to submit the article for publication.

## Results

### Properties of implants

The physical appearance and rheological properties of ABGS (rhBMP6/ABC) or PBO implants were the same. Sixty to 90 minutes after the start of preparation (blood draw), the implants were described as the following: red to deep red color, cylindrical shape, coagulated mass that detaches from the syringe wall.

### Demographics of study participants

**Fig 1:**
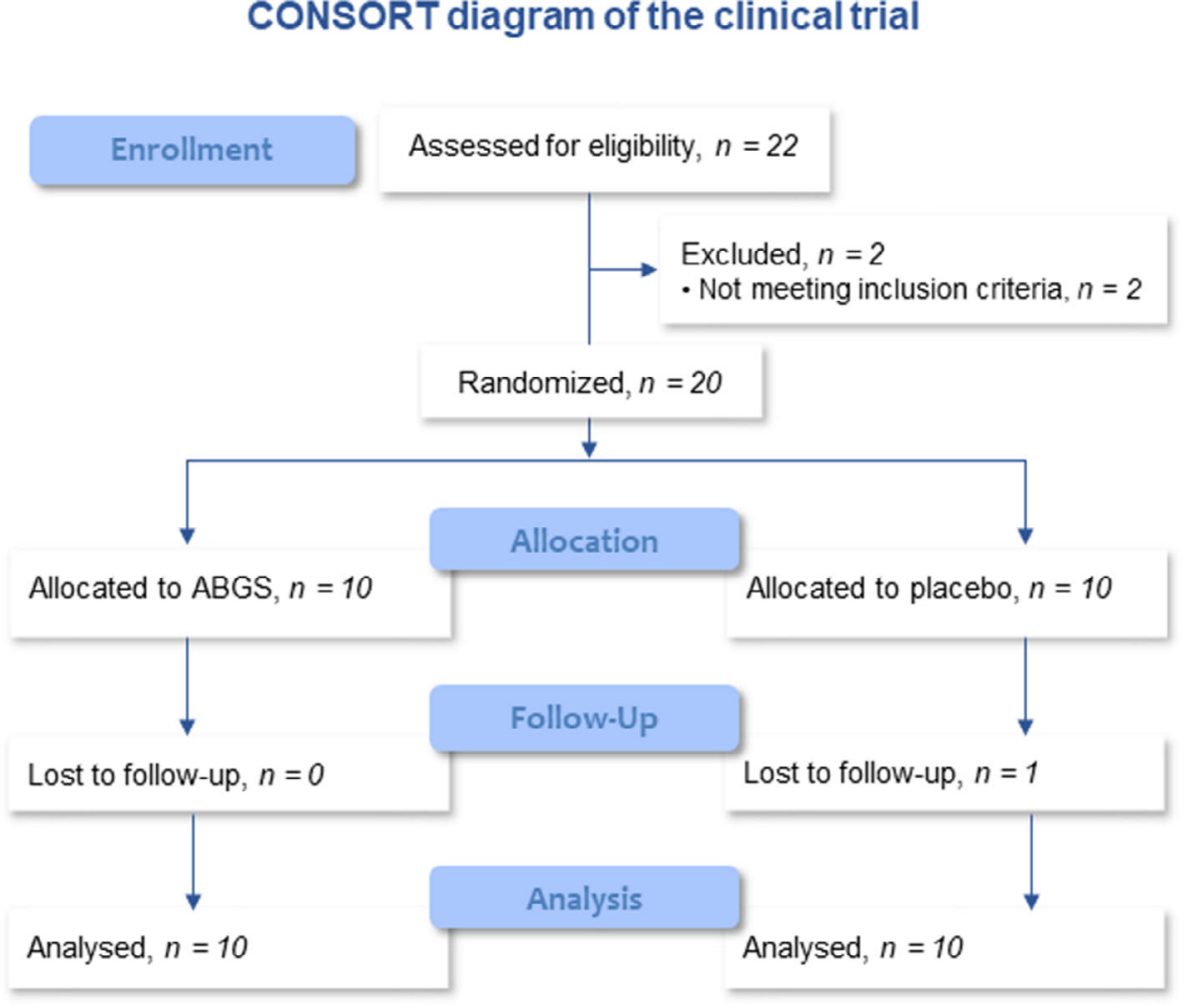
CONSORT diagram of the clinical trial.

The trial was conducted at one study site (Medical University of Vienna, Department of Orthopedics and Trauma Surgery) from June 2016 to November 2019. The study enrolled 22 participants of which 2 were found ineligible during screening; therefore, 20 participants (12 males, 8 females) received assigned treatment as per original study plan (Fig. 1; Table 1). Smoking status was recorded as 5 in the PBO group and 4 in ABGS group, respectively. The mean age of the patients was 46 years in the PBO group and 53 in the ABGS group. The range of wedge height in patients treated with ABGS was 6.8 to 15.5 mm, whereas in PBO it was between 7.4 and 15.1 mm. Patients treated with ABGS were statistically older and the wedge volume was larger compared with PBO. The trial ended as planned after the last patient completed the 24‐month follow‐up procedures. All patients but one, who missed the last follow‐up visit due to a fatal myocardial infarction, completed all follow‐up visits.

**Table 1:** Baseline Characteristics of the Treated and Analyzed Participants, According to Study Group (*t* Test for Independent Samples)

Characteristic	PBO (10 mL)	ABGS (1 mg/10 mL)	*p* Value
Patients	10	10	
Sex (male/female)	6/4	6/4	
Side (left/right)	6/4	8/2	
Age (years)	46.6 ± 5.7	53.4 ± 7.58	0.023
Height (cm)	175.5 ± 6.79	171.5 ± 12.1	0.200
Weight (kg)	91.99 ± 15.55	87.6 ± 22.6	0.319
Body mass index (kg/m^2^)	29.75 ± 3.82	29.3 ± 4.55	0.411
Length of osteotomy base (mm)	70.08 ± 7.78	70.82 ± 9.2	0.424
Medial width of osteotomy (mm)	10.90 ± 2.63	12.82 ± 4.92	0.158
Wedge volume (voxels)	71.983.90	88.044.20	0.378
Smoking status (yes/no)	5/5	4/6	—

PBO = placebo; ABGS = autologous bone graft substitute.

Plus‐minus values are means ± SD.

### Safety and adverse events

During the treatment and trial duration, no infections or adverse reactions were reported. On the other hand, for all 20 patients included in the trial, adverse events were reported, either serious (SAE) or non‐serious (AE). There was a total of 19 SAEs and the most often reported was locking plate removal, which occurred in 7 ABGS and 7 PBO patients. This was standard‐of‐care locking plate removal due with hospitalization in month 18 after HTO surgery. Additionally, there was one prolonged hospitalization per group reported that occurred as a result of an administrative error and personal reasons. In the ABGS group, one patient was hospitalized due to a fall, whereas in the PBO group, one patient had a right knee arthroplasty (contralateral) and one patient experienced a myocardial infarction (fatal) before the 24‐month follow‐up. None of these SAEs was related to the investigational drug as judged by the investigator and Independent Data and Safety Monitoring Board (IDSMB).

All of the AEs (32 events recorded in 11 patients) were rated mild to moderate and assessed as not related to the treatment by the investigator and IDSMB. In the PBO group, the following AEs were recorded: arthralgia (1×), fall (2×), pain in extremity (1×), peripheral swelling (2×), hypothyroidism (1×). In the ABGS group, the following AEs were recorded: arthralgia (2×), blood cholesterol increased (1×), headache (2×), heart rate decreased (1×), heart rate increased (1×), hypertension (6×), hypotension (1×), limb injury (2×), nasopharyngitis (3×), oropharyngeal pain (1×), peripheral swelling (1×), tremor (1×), vertigo (1×), vomiting (1×), wound necrosis (1×). ABGS group consisted of statistically older patients with previously more known underlying comorbidities.

### Pharmacokinetics of rhBMP6 and anti‐rhBMP6 antibodies

The plasma samples for PK analysis were taken from all human subjects participating in the study. RhBMP6 was detected in one sample at an early time point (15 minutes) at a concentration of 8.56 ng/mL, as assessed by a validated ELISA. For other plasma samples, no measurable amounts of rhBMP6 were detected at any time point. The blood collected before surgery and at 14 weeks post‐implantation from all subjects participating in the study did not show measurable amounts of anti‐rhBMP6 antibodies, as assessed by indirect ELISA method.

### Efficacy outcomes

#### Acceleration of bone healing

**Fig 2:**
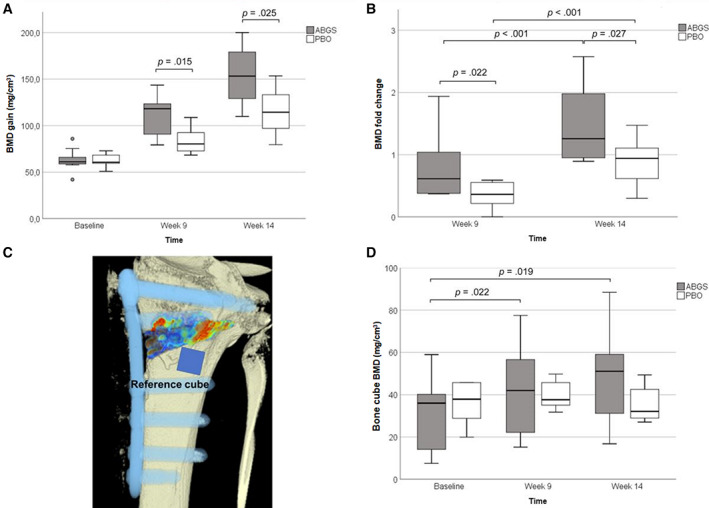
Bone mineral density (BMD) measurements. (*A*) BMD measured in the wedge of two groups of high tibial osteotomy (HTO) patients (autologous bone graft substitute [ABGS] and placebo [PBO]) from baseline (measured before the treatment) to weeks 9 and 14 after surgery. (*B*) BMD fold change at weeks 9 and 14 in patients treated with ABGS or PBO. (*C*) The location of a reference cube that served as a BMD indicator of the adjacent bone because rhBMP6 might have diffused from the wedge. (*D*) BMD values in the reference cube at baseline and weeks 9 and 14 after surgery. The classical box and Whisker's plot was used to present medians and interquartile ranges. *t* test for independent samples; ABSG *n* = 10; PBO *n* = 10.

There were significant differences in BMD gain at 9 and 14 weeks from the baseline in the ABGS group compared with PBO: 47.8 ± 24.1 versus 22.2 ± 12.3 mg/cm^3^, *p* = 0.015, and 89.7 ± 29.1 versus 53.6 ± 21.9 mg/cm^3^, *p* = 0.025, respectively (Fig. 2*A*). The fold‐percentages at 9 and 14 weeks in relation to the baseline values were also significantly higher in the ABGS group: 81.4% ± 53.3% versus 35.9% ± 18.9%, *p* = 0.022, and 148.4% ± 63.4% versus 87.4% ± 37.5%, *p* = 0.027, respectively (Fig. 2*B*). Upon adjusting for age, body mass index (BMI), and volume in voxels, there were significant differences in BMD gain at 9 weeks (*p* = 0.009) and 14 weeks (*p* = 0.020) and fold percentages at 9 and 14 weeks in relation to the baseline were with higher scores (*p* = 0.039, *p* = 0.0027, respectively) in patients treated with ABGS. The ordinary least squares (OLS) regression model showed that the PBO group compared with the ABGS group had a significantly negative impact on the increase of fold percentages after 9 weeks: β = −0.498, *p* = 0.037. In addition, if volume in voxels was higher, the increase of fold percentages after 9 weeks significantly decreased: β = −0.472, *p* = 0.038 (Supplemental Table S1).

#### Bone mineral density of the adjacent bone

To explore a potential influence of the ABGS on the trabecular bone adjacent to the osteotomy wedge, we examined the BMD status using a reference cube distally from the lower margin of the wedge having an average size of 16 × 16 × 16 voxels (1 voxel = 0.5 mm) as marked in Fig. 2*C*. CT analysis showed a significant increase in BMD in patients treated with ABGS compared with baseline values, whereas BMD in PBO‐treated patients remained unchanged as shown at 9 and 14 weeks, respectively (Fig. 2*D*).

#### Analysis of bone healing in non‐smokers versus smokers

**Fig 3:**
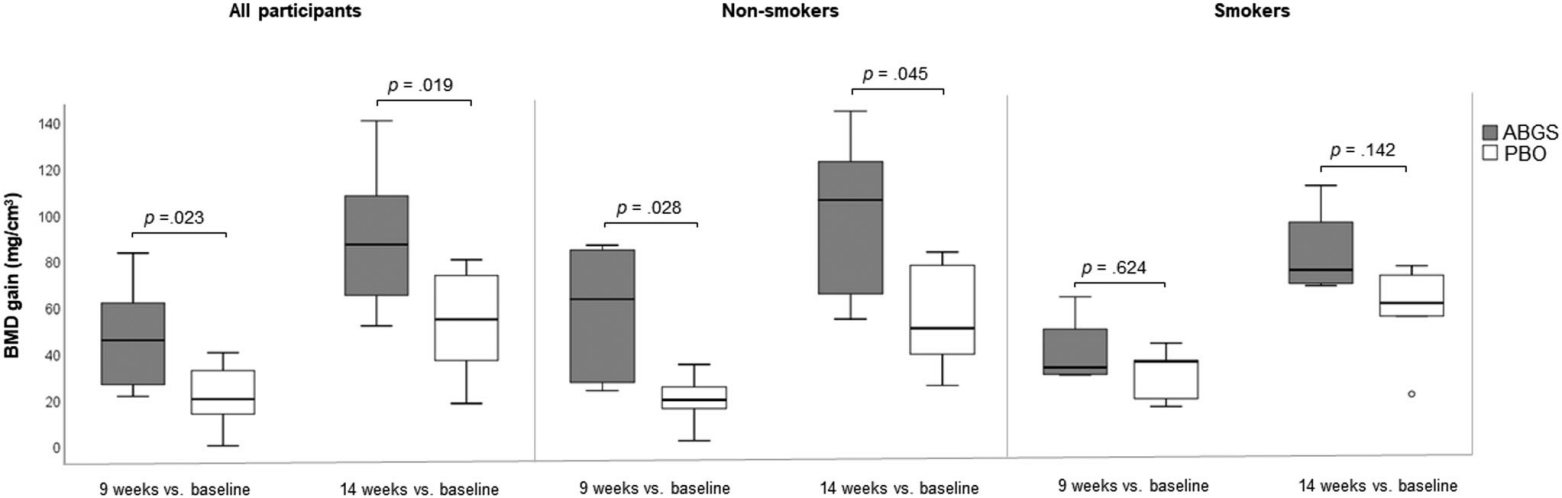
Bone mineral density (BMD) gain in autologous bone graft substitute (ABGS)‐ and placebo (PBO)‐treated patients after high tibial osteotomy (HTO) surgery in non‐smokers versus smokers (ABGS smokers *n* = 4; ABGS non‐smokers *n* = 6; PBO smokers *n* = 5; PBO non‐smokers *n* = 5) at week 9 and week 14. In the data analysis, we used non‐parametric tests (Mann–Whitney *U* test for independent specimens and Wilcoxon test for dependent specimens).

A subgroup analysis of HTO non‐smoker patients showed a robust stimulation of BMD at 9 and 14 weeks after surgery, whereas in smokers there was no difference against HTO patients treated with ABGS versus PBO. A similar finding was observed in the adjacent bone by subgroup analysis, where non‐smokers responded more favorably, whereas smokers did not. However, the radiographic analysis at months 12 to 24 showed that the ABGS‐treated group (including smokers) appears to have accelerated healing compared with the PBO group (Fig. 3; Supplemental Fig. S1).

### Pain measurement in HTO study patients

**Fig 4:**
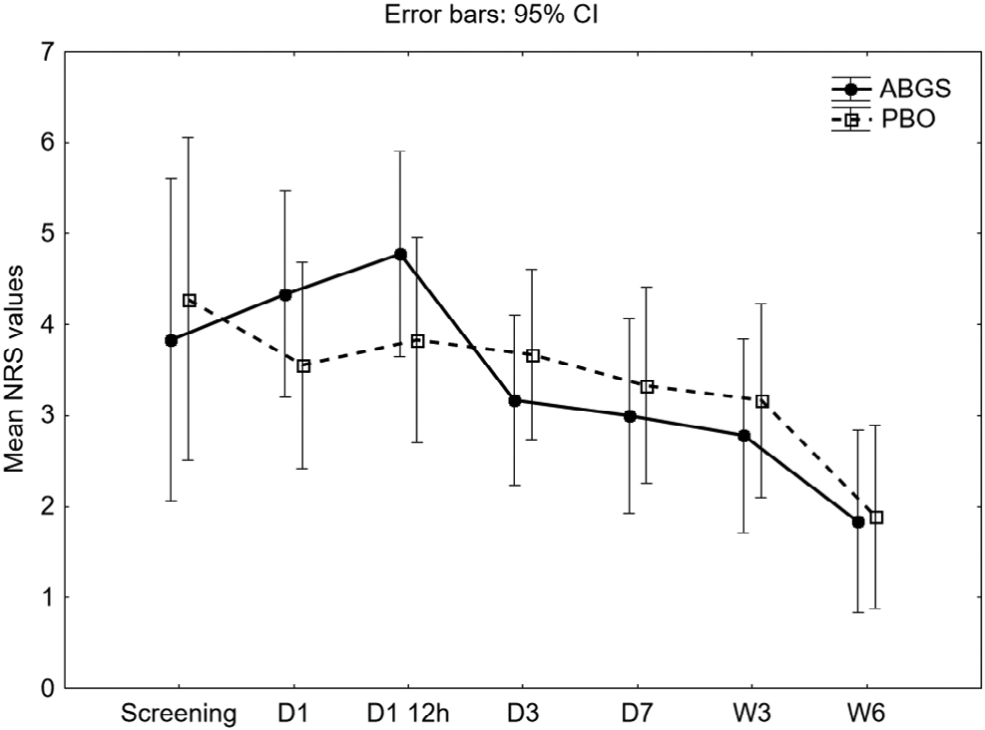
Numeric rating scale (NRS) for pain in autologous bone graft substitute (ABGS)‐ and placebo (PBO)‐treated patients after high tibial osteotomy (HTO) surgery. The values are mean ± 95% CI; *p* values for each measured time were calculated using *t* test for independent samples, while difference in dynamics between groups was analyzed with ANOVA for repeated measures. There was no significant difference between groups regarding dynamics (*p* = 0.574), whereas both groups showed significant decrease over time (*p* < 0.001). The pain level was rated as follows: no pain = 0; mild pain (nagging, annoying, interfering little with daily living activities) = 1–3; moderate pain (interferes significantly with daily living activities) = 4–6; severe pain (disabling; unable to perform daily living activities) = 7–10. ANOVA for repeated measures, ABGS *n* = 10; PBO *n* = 10. D1‐ day 1, 6h post dosing; D1 12h ‐ day 1, 12h post dosing; D3 ‐ day 3; D7 ‐ day 7; W3 ‐ week 3; W6 ‐ week 6.

The pain in both ABGS‐ and PBO‐treated patients was evaluated by a numeric rating scale (NRS).^(^ ^26^ ^)^ At 12 hours after surgery, pain was moderate and decreased to the mild pain range within 6 weeks of follow‐up. Only a few patients had severe pain, while, on average, none of the mean values from day 1 until week 6 were above the moderate pain rating from 4 to 6. In general, there was no difference between ABGS‐ and placebo‐treated patients regarding pain dynamics (*p* = 0.574), while both groups showed significant decrease over time (*p* < 0.001) (Fig. 4).

### Extended follow‐up by radiography

**Fig 5:**
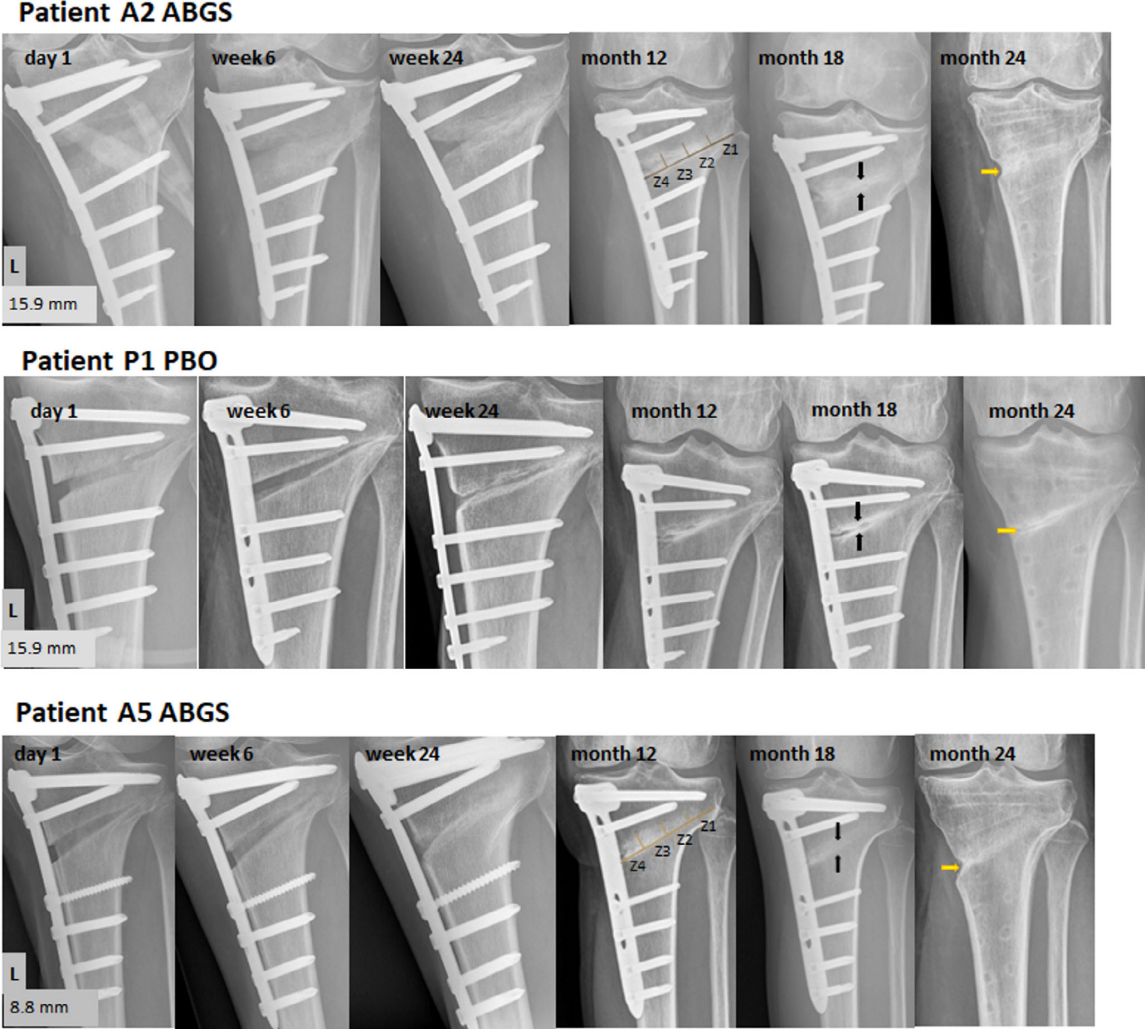
X‐ray images of isolated defect areas from 3 patients treated with autologous bone graft substitute (ABGS) or placebo (PBO) from day 1 to month 24. In patients A2 and A5 at 12 months, X‐ray zones 1 to 4 have been marked for characterization of new bone in the defect areas during X‐ray read‐outs. Black arrows shown for the defect area at month 18 indicate more pronounced BMD on X‐rays of a PBO‐treated patient compared with ABGS. However, at 12 months, still there is a gap in zone 4 (Z4) for both groups as shown on X‐ray images. At month 24, in the medial site of the gap for both groups, an incomplete cortical‐periosteal surface restoration after plate removal is indicated (yellow arrows).

**Fig 6:**
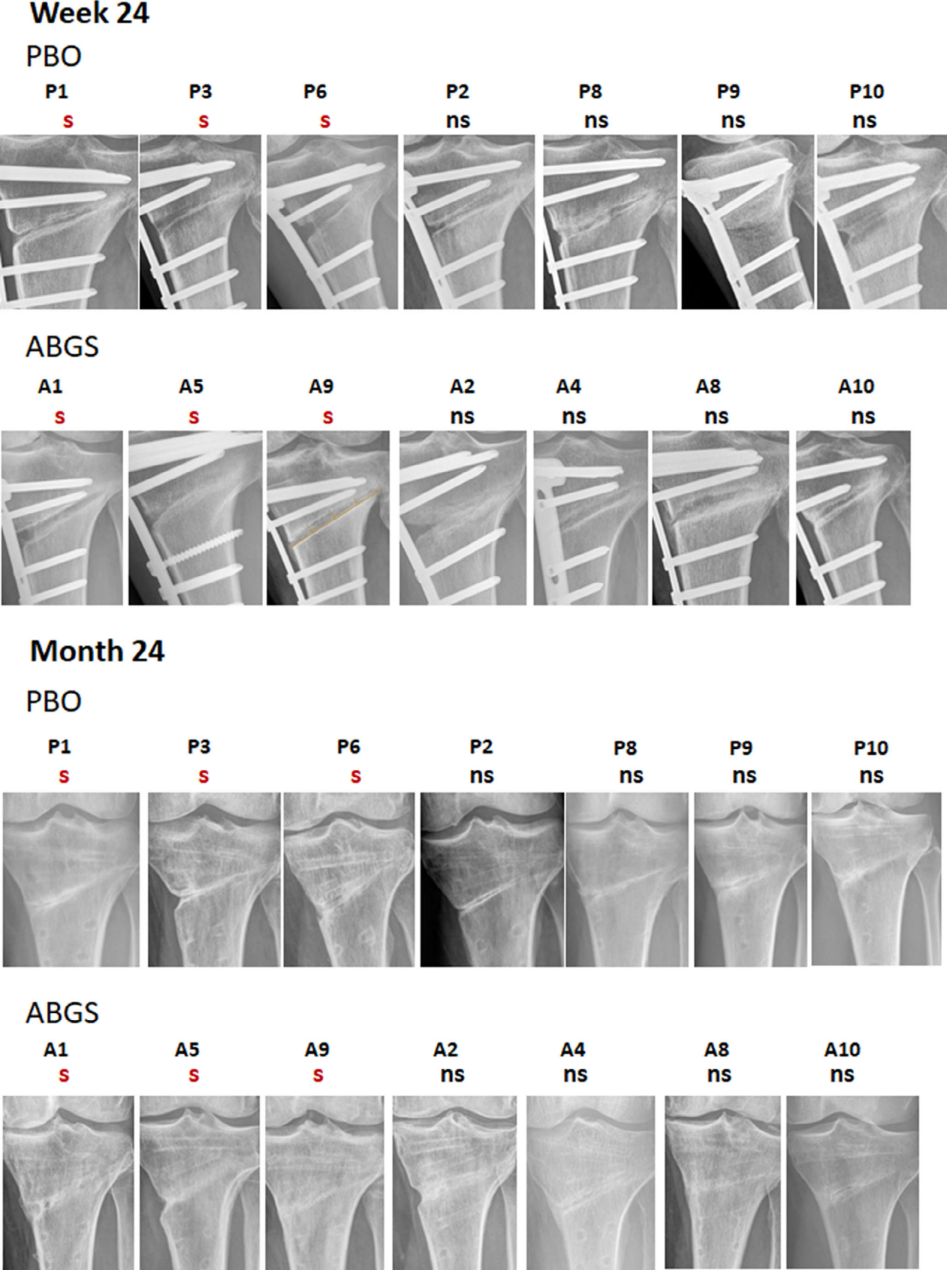
X‐ray images of isolated defect areas from 7 (3 smokers and 4 non‐smokers) high tibial osteotomy (HTO) patients per group at week 24 and month 24 treated with autologous bone graft substitute (ABGS) or placebo (PBO). At week 24, patients treated with ABGS showed more new bone formation than those treated with PBO irrespective of smoking status. At 24 months after the plate removal, the differences between the two treatment groups were maintained in favor of the ABGS‐treated patients with advanced repair and remodeling in non‐smokers (ns) compared with smokers (s).

Patients treated with ABGS showed increased areas of new bone formation at 6 and 24 weeks in the wedge compared with PBO treatment. The results have been evaluated by two orthopedic surgeons and a radiologist with substantial intra‐observer variation of more than 70% (data not shown). Subsequently, serial radiographs of patients treated with ABGS from day 1 to month 24 showed a gradual closing of the tibial wedge by newly formed bone with a pronounced remodeling compared with PBO‐treated patients (Fig. 5). At month 12, the majority of the defects in ABGS‐treated patients were closed, whereas PBO‐treated patients still had partial defects (Figs. 5 and 6). There was no visible sclerosis and no signs of bone resorptive rough surfaces in patients treated with ABGS, whereas some sclerotic bone surfaces were found in PBO‐treated patients (Fig. 6). The cortical bone was not yet fully restored and mineralized in any of the 20 patients, suggesting that rigid fixation of the defect with the locking plate may have interfered with the formation of the medial cortex.

The results demonstrate that rhBMP6/ABC (ABGS) significantly accelerated bone healing over PBO and that the wedge defect was filled with trabecular bone as determined by CT readouts of BMD at 9 and 14 weeks and radiographic image read‐outs from week 6 to month 24.^(^ ^27^, ^28^ ^)^

## Discussion

To the best of our knowledge, this is the first clinical study in which a BMP‐based biological osteogenic therapy was evaluated against placebo in the trabecular bone surface of an orthopedic indication. We show that ABGS that contains rhBMP6 applied within autologous blood (ABC) and administered as a coagulum is capable of inducing new bone formation with an accelerated rate compared with PBO. This bone‐forming effect was demonstrated in an open wedge‐defect gap of patients who elected to undergo a HTO procedure for varus deformity of the knee to delay the progression of osteoarthritis and medial osteoarthritis.

This ABGS‐HTO clinical trial was randomized, controlled, and quadruple‐blinded from the perspective of patients, pharmacists, surgeons, and evaluators. The demographics of the study involved patients with an equal sex distribution, BMI, wedge‐gap volume, and smokers, except that the ABGS group was statistically older than the placebo group. The baseline BMD was not measured because patients with osteoporosis were excluded from the study. However, the initial BMD was determined in the healthy bone close to the wedge and then at a later time. Medical history of use of anti‐osteoporotic drugs was recorded as an exclusion criterion. ABGS was found to be safe because there were no swelling, redness of the skin, edema, distant soft tissue ossification, and no observable systemic side effects over the 24‐month follow‐up. Administered rhBMP6 was detected in circulation of only one patient immediately after implantation (at 15‐minute time point), and there were no antibodies against rhBMP6 detected at 14 weeks after implantation. The safety profiles as monitored by serious adverse events (SAEs) were comparable in both groups and not related to the drug as judged by the investigator and IDSMB. Although there was a higher proportion of AEs in the ABGS group (25 versus 7), the reported AEs are previously known comorbidities for this observed patient group. The adverse events were not clinically significant and were not related to the investigational therapy applied as judged by the investigator and the IDSMB. These comorbidities were determined at screening and presented no exclusion criteria.

The accelerated bone healing was significantly higher in the treatment group, as determined by BMD measurements in the wedge‐defect gap by CT, when compared with PBO from the baseline at week 9 (*p* = 0.009) and week 14 (*p* = 0.020) after adjusting for age, BMI, and volume in voxels. Radiographic analysis at 6 and 24 weeks showed a gradual closing of the tibial wedge by the newly formed bone with a marked increase from inside the wedge defect toward the outside in both groups. There were no visible osteolytic/sclerotic lesions in any of the defects treated with ABGS, whereas more sclerotic bone surfaces were found in PBO‐treated defects. Extended serial radiographic analyses showed that at week 24, none of the patients fully closed the gap, but at month 12 the majority of the defects in the ABGS‐treated group were closed, whereas in the placebo‐treated patients the defects remained partially open. The newly formed bone underwent remodeling with a faster rate in patients treated with ABGS compared with PBO. However, the cortical‐periosteal bone surface was not yet fully restored with defined cortices in any of the 20 patients examined at month 18, suggesting that stabilization of the defect with the locking plate might have interfered with the formation of the cortex at the periosteal surface. It is likely the cortices will be restored upon removing the plate and subsequently by mechanical loading. In this regard, after the plate removal, the majority of ABGS‐treated patients at month 24 showed a substantial cortical‐periosteal bone surface restoration (Fig. 6). Examination of BMD adjacent to the tibial osteotomy defect using a reference cube distally from the lower margin of the wedge showed a significant increase in patients treated with ABGS compared with baseline values at weeks 9 and 14, whereas the BMD in PBO‐treated patients remained unchanged. This distal biological osteogenic response remains to be explored further in patients compromised with metabolic insults like diabetics or osteopenia and in those undergoing knee endoprosthesis replacement. Interestingly, although there were 4 smokers in the ABGS‐treated group and 5 in the PBO group, ABGS therapy did not accelerate bone healing at the early stages in smokers. However, based on X‐ray images (Fig. 6), ABGS therapy resulted in a better outcome irrespective of smokers or non‐smokers compared with PBO by months 12 and 24, suggesting that smoking is related to an increased risk of delayed healing.^(^ ^25^, ^29^, ^30^ ^)^

Two BMP‐based osteogenic biologics, rhBMP2 soaked in an absorbable collagen sponge (InFuse) and rhBMP7 mixed with a bone collagen particulate (OP1‐Putty), have been approved for acute and open diaphyseal shaft fractures and tibial non‐unions, respectively, where autograft is not feasible. Both devices employ high doses of rhBMP (3.7 to 12 mg) presumably to overcome the Noggin inhibition and the immune response elicited by the use of animal‐derived collagen as a carrier.^(^ ^31^ ^)^ Furthermore, because of their weak binding to collagenous carrier, rhBMP2 and rhBMP7 are released readily from the implants at the local site and cause sclerosis and aberrant soft tissue ossification. On the other hand, the ABGS device examined here uses an autologous blood coagulum, a physiological carrier, to deliver rhBMP6. Furthermore, unlike rhBMP2 and rhBMP7, rhBMP6 binds weakly to Noggin, a BMP antagonist predominantly found in bone.^(^ ^11^ ^)^ RhBMP6 binds tightly to fibrin‐mesh‐work and plasma proteins, being released slowly as an intact protein in accordance with the resolution of coagulum at the implant site.^(^ ^32^ ^)^ We have shown that ABGS implants are osteoinductive, whereas ABC implants per se are not, as examined in the rat subcutaneous bone formation assay.^(^ ^9^ ^)^ The cumulative release of rhBMP6 from ABGS was sustained *in vitro* over 2 weeks and did not exceed 9%.^(^ ^9^ ^)^ The rhBMP6 released from the ABGS implant was intact and biologically active as measured by ELISA and the BRE‐reporter assay using mouse C2C12 transfected cell line. RhBMP6 from the implant is likely released *in vivo* during the dissolution of ABC through cellular processes and enzymatic activity at the local site.

The optimal effective dose (100 μg/mL) was chosen for a single administration based on several preclinical studies using small and large animal models for fracture healing and posterolateral lumbar fusion.^(^ ^6^, ^7^, ^9^ ^)^ Since we employed autologous carrier in animals as well as in human patients instead of xenogeneic animal‐derived collagen as a carrier, the effective dose is translatable from preclinical models to human trials. Avoidance of animal‐derived collagen helped to minimize the inflammatory response at the local implant site and the systemic immune responses, and thus prevented edema, erythema, and tenderness. Because there have not been any reports of rhBMP‐based osteogenic device to promote bone healing in trabecular bone surfaces, the evaluation of rhBMP6 described here represents the first of such findings. The limitations in the trial include a small number of patients in whom the delay of medial osteoarthritis was not explored by the long‐term follow‐up to compare the effects of accelerated healing in HTO wedge defect of ABGS compared with PBO. Also, the study was not designed to address the potential progress of the knee osteoarthritis as a relevant clinical outcome. This should be explored during further clinical development.

The successful outcome of ABGS observed in our HTO clinical trial may likely provide a basis for the evaluation of ABGS against revision surgery after total hip and knee replacement, trabecular bone defects in postmenopausal women, and diaphyseal bone non‐unions in healthy adult and neurofibromatosis type I (NF‐1) patients. It remains to be explored whether ABGS treatment may be helpful in delaying the medial osteoarthritis.

## Disclosures

HO, LG, and SV have an issued patent US8197840 and licensed to Genera Research (GR). RW is a consultant for Pfizer, Stryker, Takeda, Depuy Synthes, and Zimmer Biomet. HO is an employee of Genera Research. TKS is an employee of perForm Biologics and has received grants from perForm Biologics during the study. MP, SM, LG, TBN, SV, CC, HO, CT, and RW collaborate on the development of a new drug for bone repair under the consortium of partners funded by EU HORIZON2020 (GA No 779340 [OSTEOproSPINE]).

## Peer Review

The peer review history for this article is available at https://publons.com/publon/10.1002/jbmr.4107.

## Supporting information


**Supplemental Materials and Methods**

**Supplemental Fig. S1.**

**Supplemental Table S1.** Ordinary Least Squares Regresstion (OLS)
