# Supplementary material for: Recombinant Human BMP6 Applied Within Autologous Blood Coagulum Accelerates Bone Healing: Randomized Controlled Trial in High Tibial Osteotomy Patients
Source: J Bone Miner Res. 2020 Jul 2;35(10):1893–903. doi: 10.1002/jbmr.4107 (PMC7689741; doi:10.1002/jbmr.4107)
Supplement: Supplementary file 1 — Supplemental Materials and Methods [file JBMR-35-1893-s001.docx]

**Supplemental Materials and Methods**

Investigational medicinal product

Genera Research, a biotech company, was responsible for manufacturing of rhBMP6 and PBO, their analysis and activity testing according to GMP principles, including the drug substance and drug product containing rhBMP6 as active pharmaceutical ingredient. All data generated were presented in regulatory compliant documentation and submitted to regulatory authorities that investigated the available data and issued their approvals for use of the product in human clinical trials. Among numerous used assays for determining drug substance and drug product properties, characteristics and stability, the activity of rhBMP6 was verified by the *in vitro* testing - mouse C2C12-BRE-Luc BMP reporter cell assay, as described in Grgurevic et al. JBMR Plus 2019 (ref. 9) as well as by the *in vivo* subcutaneous ectopic bone formation assay in CD1 male mice.

Intervention

The operation started without a tourniquet. A standard approach to the medial proximal tibia was performed. After release of the medial collateral ligament the osteotomy was performed with an oscillating saw. The tourniquet was activated just before starting with the osteotomy. The osteotomy was opened according to the preoperative planning to achieve optimal correction of the limb alignment. The locking plate (TomoFix, Synthes) was used to stabilize the osteotomy gap. The periosteum was adapted with sutures underneath the plate leaving a small window for administration of the therapy. Before administration of the study device the lower leg was wrapped with a rubber bandage, the tourniquet was opened and after 10 minutes the bandage was removed. The solid natural coagulum created by this technique was sucked out of the osteotomy gap in order to achieve an empty and dry gap. The syringe with the investigational therapy was given to the orthopaedic surgeon by the pharmacist into the sterile field. The therapy was injected from a syringe into the gap and the small window was closed with soft tissue flap sutures and a haemostyptic (Tabotamp Fibrillar, Ethicon, Johnson and Johnson, New Brunswick, NJ, USA). Drains without suction were applied in the subcutaneous layer. Subcutaneous and cutaneous closures were performed.

Clinical study design

As there were no BMP-based products tested for this indication, patient enrolment was gradual. The time interval of enrolment from one patient to another had a 7-day observation period that included daily clinical (local and systemic) evaluation in-hospital stay and safety laboratory assessments at days 1, 3 and 7 prior to discharge. Phase II proceeded upon getting an approval from Independent Drug Safety Monitoring Board (IDSMB) who was responsible for the overall assessment of safety after 6 weeks of follow-up based on clinical, laboratory and x-ray data from the first 6 patients. This stage enrolled a total of 14 patients, 5 of whom were randomized to ABGS and 9 to PBO.

Sample size determination

For Phase I of the trial, it was determined that 6 patients, 5 randomized to ABGS and 1 to placebo were sufficient to detect any relevant, systemic or local, safety signals based on a large body of non-clinical data (PD & PK studies, GLP safety and local tolerance studies, and non-GLP efficacy studies in animals) and the use of ABC as a carrier. In total, sample size (N=20, randomized 1:1 to ABGS and placebo) was determined with the intention to enable detection of a clinically relevant bone healing-acceleration effect.

The two-sided alpha 0.1 was assumed comparable and constant variance at two groups and time points expressed as relative standard deviation of 100%. This difference was considered as practically relevant in terms of acceleration of metaphyseal bone healing. No dropouts were expected considering the specific indication and standard of care for these subjects. This sample size was also considered sufficient to detect the level of systemic exposure to rhBMP6 after ABGS administration into the HTO wedge defect.

Safety outcome measures

MedDRA versions 19.0, 19.1, 20.0, 20.1, 21.0, 21.1, 22.0 and 22.1 were used for the coding of adverse events in the cumulative study period. Serious adverse events like hospitalization related to elective surgery for HTO plate removal upon healing of the osteotomy gap, was not considered a serious adverse event, although they were all reported as such. Any adverse events and serious adverse events occurring at any given time during the trial (clinical, laboratory, radiological), local or systemic, were regularly evaluated by the independent monitor, pharmacovigilance provider and Independent Data and Safety Monitoring Board (IDSMB). All enrolled patients were followed-up for 24 months. The time for the removal of the plate material was at 18 months.

PK outcome measures

In addition to safety monitoring, during the first 24 hours, blood samples were collected for PK assessment. Plasma samples were obtained immediately before treatment (time 0) and then at 15, 30, 45, 60 min and 1.5, 2, 4, 6, 12 and 24-hours post-dose and stored at -80 °C until central analysis. The detection and quantification of total rhBMP6 in the plasma samples was performed by an in-house validated sandwich ELISA method using human BMP6 DuoSet ELISA Development kit (DY507; R&D Systems) with the clinical batch of rhBMP6 (no. F15227, Baccinex SA) as the internal reference standard. Plasma Noggin values were determined by the commercially available ELISA kit (Nordic Biosite EKH2102) in undiluted samples from the time point 0.

Efficacy outcome measures

*Acceleration of bone healing*

For estimation of BMD from the intensity values a Model 3CT Calibration Phantom composed of 5 rods of reference material embedded in the plastic base was used, with the transferred segmentation mask acquired at baseline (Mindways, Austin, TX, USA). The CT calibration phantom, calibrated against liquid K_2_HPO_4_/water solutions, was placed under the osteotomy wedge for conversion of CT Hounsfield number to equivalent K_2_HPO_4_ density, yielding a linear relation between Hounsfield units (HU) and the dipotassium phosphate (K_2_HPO_4_) equivalent to BMD. It was assumed that K_2_HPO_4_ was identical to the bone ash density in agreement with a previous study on QCT using different calibration phantoms^(^[^1^](#_ENREF_1)^)^. Each HU value in the CT image was converted to the volumetric BMD (vBMD, mg/cm^3^) using the phantom-derived linear regression equation. Due to the assumed linear relationship between HU and BMD, voxels with radiopaque density smaller than water (i.e. fat) have a negative BMD. We corrected this fat error by setting all negative BMD values to 0.

Due to the fact that there was blood in the osteotomy wedge, a semi-automatic segmentation based on a supervoxel algorithm was used. An automatic algorithm would fail because it would not be able to distinguish between bone marrow and blood, since they have similar intensities (Hounsfield units). The supervoxel algorithm grouped similar voxels in the image into supervoxels (i.e. generating 3D puzzle pieces) – these voxels were segmented manually, all of them resulting in the segmented wedge. The corrected vBMD value for each wedge for the initial time point and every follow-up of each subject was computed by sampling all voxels within the respective segmented region (i.e. all voxels inside the wedge). Finally, the vBMD of osteotomy wedge was determined by averaging the mean vBMD values and standard deviation (SD) for every time point (i.e. baseline, first follow-up and second follow-up). The local changes of the osteotomy wedge were also compared in a voxel-vise manner for illustration^(^[^2^](#_ENREF_2)^)^. Based on the segmentation mask of the manually segmented osteotomy wedge, the local change of the wedge was compared voxel-vise and the changes in BMD were displayed. Measure of therapeutic effect was defined as percentage of the defect filled with newly formed bone, based on CT assessments performed at weeks 9 and 14 post-surgery^(^[^3^](#_ENREF_3)^,^[^4^](#_ENREF_4)^)^.

*Segmentation of the splint and correspondence between time points*

The internal fixation device (splint) is a device that emerged under the trade name of TomoFix (Synthes GmbH; Solothurn, Switzerland). To extract the splint in the MDCT images we used an automated segmentation algorithm based on a simple threshold that was combined with registration to match the positions of the splint during two or more time points (follow-up). We used a ridged registration based on sum of squared differences to find correspondences between the two time points. The segmented osteotomy wedge mask at baseline was transferred accordingly to the corresponding follow-up image.

*Reference Area*

For comparing the vBMD within the tibia, we annotated a reference cube with an isotropic voxel size of 16 voxels, placed adjacent under the wedge. We used the same transformation values to map the reference cube masks onto the follow-up examinations. We also determined the vBMD inside the reference area in the same manner as previously described for comparison.

Statistics

Smirnov-Kolmogorov test was used to assess data distribution using appropriate parametric analysis. T-test for independent samples was used to analyse bivariate differences between the groups (ABGS and PBO). Additionally, we separated groups to smokers and non-smokers and analyse their data with non-parametric tests (Mann-Whitney U test for independent specimens; and Wilcoxon test for dependent specimens) since they had mainly non-parametric distribution of investigated numeric values. Analysis of covariance (ANCOVA) was used afterwards to statistically control the possible effects of significant confounding variables on differences between investigated groups. Preliminary checks were conducted to ensure that there were no violation of the assumptions of normality, linearity, homogeneity of variances, homogeneity of regression slopes, and reliable measurement of the covariate. Analysis of variance for repeated measures (RM-ANOVA) has been made to assess differences in NR scale for pain regarding measuring time and investigated groups (ABGS and PBO). Ordinary least square regression (OLS regression) was performed to evaluate influence of investigated groups, age, BMI and volume in voxels on fold difference after 9 and 14 weeks and baseline level. All P values below 0.05 were considered significant. Statistical softer IBM SPSS Statistics version 25.0 was used in all statistical procedures. Data was blindly analysed, and radiological outcomes were assessed by an independent expert panel of two orthopaedic surgeons and one radiologist.

References

1. Suzuki S, Yamamuro T, Okumura H, Yamamoto I. Quantitative computed tomography: comparative study using different scanners with two calibration phantoms. The British journal of radiology. Nov 1991;64(767):1001-6.

2. Nemecek E, Chiari C, Valentinitsch A, Kainberger F, Hobusch G, Kolb A, et al. Analysis and quantification of bone healing after open wedge high tibial osteotomy. Wiener klinische Wochenschrift. Sep 9 2019;131(23-24):587-98.

3. Cann CE. Quantitative CT for determination of bone mineral density: a review. Radiology. Feb 1988;166(2):509-22.

4. Mucha A, Dordevic M, Hirschmann A, Rasch H, Amsler F, Arnold MP, et al. Effect of high tibial osteotomy on joint loading in symptomatic patients with varus aligned knees: a study using SPECT/CT. Knee surgery, sports traumatology, arthroscopy : official journal of the ESSKA. Aug 2015;23(8):2315-23.

**Supplemental figure 1.** Bone gain in HTO patients treated with ABGS or PBO in non-smokers vs. (ABGS smokers n=4; ABGS non-smokers n=6; PBO smokers n=5, PBO non-smokers n=5) within the bone reference cube adjacent to the distal margin of the tibial wedge. In the data analysis we used non parametric tests (Mann-Whitney U test for independent specimens; and Wilcoxon test for dependent specimens). The classical Box and Whisker’s plot was used to present medians and interquartile ranges. In other figures the mean values and 95% confidence interval was used.


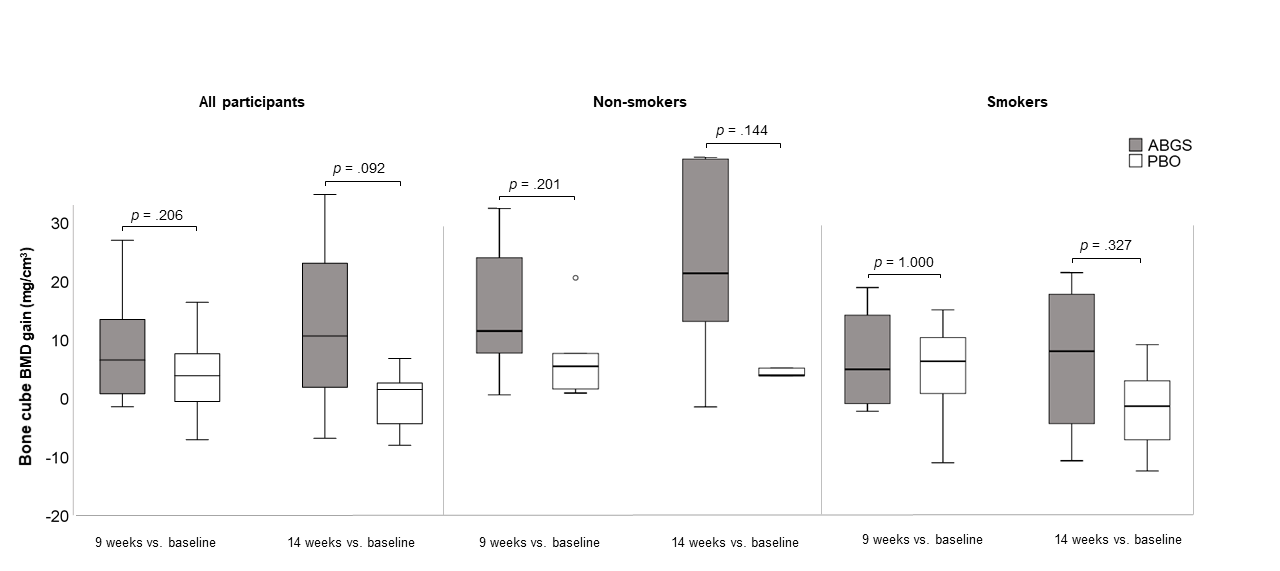


**Supplemental table 1.** Ordinary Least Squares regression (OLS)

| Model Summary | | | | |
| --- | --- | --- | --- | --- |
| Model | R | R Square | Adjusted R Square | Std. Error of the Estimate |
| Fold (%) 9 w vs. baseline | 0.730^a^ | 0.533 | 0.366 | 0.3615 |

a. Predictors: (Constant), volume in Voxels, Age (years), BMI (kg/m^2^), Group, Gender

|  | **Unstandardized Coefficients** | | **Standardized Coefficients** | **t** | **P** | **95.0% Confidence Interval for B** | |
| --- | --- | --- | --- | --- | --- | --- | --- |
|  | **B** | **Std. Error** | **Beta** |  |  | **Lower Bound** | **Upper Bound** |
| (Constant) | 0.982 | 1.476 |  | 0.665 | 0.517 | -2.185 | 4.149 |
| B vs. A group | -0.441 | 0.192 | -0.498 | -2.298 | 0.037 | -0.853 | -0.029 |
| Age (years) | 0.015 | 0.013 | 0.256 | 1.123 | 0.280 | -0.014 | 0.044 |
| Female vs. Male | -0.135 | 0.215 | -0.150 | -0.628 | 0.540 | -0.597 | 0.327 |
| BMI (kg/m^2^) | 0.005 | 0.023 | 0.043 | 0.196 | 0.847 | -0.045 | 0.054 |
| Volume in Voxels | -5.419E-06 | 0.000 | -0.472 | -2.284 | 0.038 | 0.000 | 0.000 |

a. Dependent Variable: Fold (%) 9w vs. baseline
